# Determining total phenolic content and total antioxidant capacity of loquat cultivars grown in Hatay

**DOI:** 10.4103/0973-1296.59959

**Published:** 2010-02-13

**Authors:** A. Aytekin Polat, Oğuzhan Çalişkan, Sedat Serçe, Onur Saraçoğlu, Cemal Kaya, Mustafa Özgen

**Affiliations:** *Department of Horticulture, Faculty of Agriculture, Mustafa Kemal University, Antakya, 31040, Hatay, Turkey*; 1 *Department of Horticulture, Faculty of Agriculture, University of Gaziosmanpasa, Tasliciftlik, 60240, Tokat, Turkey*; 2 *Department of Food Engineering, Faculty of Agriculture, University of Gaziosmanpasa, Tasliciftlik, 60240, Tokat, Turkey*

**Keywords:** Antioxidant, *Eriobotrya japonica*, ferric reducing ability of plasma, fruit, phenolics, pomology, TEAC

## Abstract

Several fruit characteristics of five loquat (*Eriobotrya japonica* (Thunb.) Lindl.) cultivars/selections grown in Dörtyol, Hatay, Turkey were investigated in 2008. The cultivars/selections included ‘Baduna 5’, Güzelyurt 1, ‘Hafif Çukurgöbek’, ‘Ottaviani,’ and Type 1. The characteristics evaluated included fruit weight, width, length, seed number and weight, flesh/seed ratio, total soluble solids (TSS), pH, acidity, total phenolic (TP) content, and total antioxidant capacity (TAC), determined by the ferric reducing antioxidant power (FRAP) assay. The analyses were conducted by three replicates, with 30 fruits in each replicate. The results indicated that there were significant differences among the cultivars, for all the traits tested. For example, ‘Hafif Çukurgöbek’ and ‘Ottaviani’ had smaller fruits than others, although ‘Hafif Çukurgöbek’ had heavier seeds. The flesh/seed ratio was the highest in Type 1, while ‘Hafif Çukurgöbek’ had the highest pH and high soluble solids. ‘Baduna 5’ and ‘Hafif Çukurgöbek’ had the highest acidity. The TP ranged from 129 (‘Baduna 5’) to 578 (‘Hafif Çukurgöbek’) mg gallic acid equivalent (GAE)/kg fresh fruit (fw). ‘Hafif Çukurgöbek’ also had the highest FRAP mean (12.1 mmol Trolox Equivalent (TE)/kg fw). The results suggest that loquat cultivars have a variable range of TP content and a relatively high total antioxidant capacity, which is crucial for human health.

## INTRODUCTION

The loquat (*Eriobotrya japonica* Lindl. *Rosaceae, Maloideae*) is an evergreen fruit species grown in the subtropical zone, especially in the citrus-growing regions.[1] However, loquat has more specific environmental requirements than citrus.[2] Being different from other fruit species, the blossoming period of the loquat trees occurs in winter and fruits are harvested 152-189 days after the full bloom.[3] Thus, the fruits of the loquat can be sold at a higher price in spring, as there are few competitive fruits in the market except for strawberry, green plum, and green almond. The loquat is also rich in vitamins A, B, and C, mineral substances, salts, and sugar, making it important for nourishment during the low period of the fresh fruit season.[4]

The world loquat production was 566.031 tons and China stands out as the main producer and Spain as the prime exporter.[4] Turkey is one of the most important producers of loquat in the world. In 2006, Turkey ranked fourth in production in the world, with approximately 12,310 t. Until the last decade, loquat growing was carried out only in isolated home gardens for local consumption. After its economical value had been realized, demand for its commercial production rapidly increased. The total production in 1980 (3,000 t) increased more than three-fold by 1990 (9,000 t), four-fold by 2006, and reached about 12,310 t, which was produced from 243,429 trees.[5]

In Turkey, loquat is produced in certain parts of the Mediterranean, Aegean, and Black Sea regions, which have a subtropical climate. The Mediterranean region has the most suitable ecological conditions for growing this fruit;[6] in 2006, 91.2% of the plantations were located in the Mediterranean region lying in the southern part of the country, with 97% of the total loquat production of Turkey being from the coastal zone. Specifically 91.2, 4.4, 4.2, and 0.2% production was in the Mediterranean, Aegean, Black Sea, and Marmara, respectively. In addition, from that study, 97, 2.0, 0.9, and 0.1% of the total number of loquat trees were found growing in these four areas, respectively. Loquat production in the Mediterranean region has increased by 300% in the last decade. In this region, Antalya ranks first and Hatay ranks fourth, in the production of loquat trees.[4,5]

While loquats meet the demand when there are not many fresh fruits available in the market, it also has an important place in nutrition, with its high vitamin A, B, and C content, minerals, and sugars.[7] Some of these phytochemicals, which act as antioxidants, have increasingly been seen to help optimize human health by neutralizing the harmful free radicals in the body. These antioxidants reduce the oxidative damage to cells that can lead to cancer, heart disease, and other degenerative diseases.[8] It has been seen that loquat has a high ability to scavenge free radicals and suppress LDL oxidation.[9]

The antioxidant activity of plant parts is mainly contributed by the constituent phenolic and flavonoid compounds. Consumer acceptance of the nutraceutical aspects of natural foods such as fruits and vegetables has increased significantly in the past few years. There is also an increase in the acceptance of formulated foods containing compounds extracted from fruits and vegetables.[10]

Although it is well known that loquats are a good source of beta-carotene[11,12] the diversity of phytochemicals within this species has not been investigated in detail, primarily because most breeding programs aim at improving the yield, altering the season, and resistance to biotic and abiotic factors. Hence, we decided to investigate the variation in pomological characteristics, phenolics, and antioxidants among several genotypes of loquat so that superior genotypes could be identified and used for further improvement.

## MATERIALS AND METHODS

### Chemicals

All the chemicals used were purchased from the Sigma and Aldrich Company and were of analytical grade.

### Plant materials

The study was conducted with fruits from five loquat cultivars/selections grown at Mustafa Kemal University, Agriculture Faculty, Horticulture Department, Dörtyol Experimental Station. The cultivars tested included ‘Baduna 5’, Güzelyurt 1, ‘Hafif Çukurgöbek’, ‘Ottaviani’, and Type 1. In 1997, on the orchard site, five loquat cultivars were budded onto seedlings and planted at a distance of 6 × 6 m. Standard cultivation methods with drip irrigation systems were applied.[13]

In 2008, the fruits were harvested at their fully mature stage from three replicates having 30 fruits in each replicate. Fruit weight (total, flesh and seed weights) was measured with a scale sensitive to 0.01 g (Precisa, ×B 2200 C). Flesh/seed ratio was calculated from these values. Fruit size determinations were made using a digital compass (BTS, 0-150 mm). Soluble solids were determined with a hand-held refractometer (N.O.W., 0-32% Brix) and pH was determined by a pH-meter. Acidity (expressed as % citric acid) was determined by titrating with 0.1 N NaOH to an endpoint of pH 8.10. The soluble solid/acidity ratio was calculated. The seed number was also determined in each fruit.

For the TP and TAC analyses, harvested fruit samples were frozen and stored at −20°C until analyzed. After thawing to room temperature, triplicate 100 g lots of loquat fruits from each cultivar were homogenized in a blender. All triplicates were screened for their total phenolic contents and antioxidant capacity following a single extraction procedure.[14] For this procedure, 3 g aliquots of each homogenate were transferred to polypropylene tubes and extracted with 20 mL of extraction buffer containing acetone, deionized water, and acetic acid (70:29.5:0.5 v/v), for one hour.

The sample total phenolic contents were measured according to Singleton and Rossi[14] with slight modifications. To determine the levels of TP, 1 mL of each extract was combined with Folin-Ciocalteu's phenol reagent and water 1:1:20 (v/v) and incubated for eight minutes, followed by the addition of 10 mL of 7% (w/v) sodium carbonate. After two hours, the absorbance of each was measured at 750 nm. The values of TP were estimated by comparing the absorbance of each with those of a standard response curve generated with gallic acid. Results were expressed as μg gallic acid equivalents on a fresh weight (fw) basis (GAE/g fw).

For obtaining the antioxidant capacity, the FRAP (ferric reducing antioxidant power) method was conducted according to Benzie and Strain.[15] To conduct the assay, a 2.95 mL aliquot of a FRAP reagent, a mixture of 0.1 mol/L acetate buffer, 10 mmol/L TPTZ, and 20 mmol/L ferric chloride (10:1:1 v/v/v), were combined with 50 μL of acetone fruit extract. To determine the antioxidant capacity of the samples, the absorbance values were compared with those obtained from the standard curves of trolox (10-100 μmol/L). The antioxidant capacity values were expressed as mmol trolox equivalent in kg fresh weight basis (TE)/kg fw.

Data were analyzed using SAS software and procedures.[16] Means and standard deviations were calculated using the TABULATE procedure. Analysis of variance tables were constructed using the Least Significant Difference (LSD) methods at 0.05. Correlation coefficients and their levels of significance were calculated using the CORR procedure.

## RESULTS AND DISCUSSION

The results indicated that there were significant differences among cultivars/selections for all the variables tested [Table 1]. For example, ‘Hafif Çukurgöbek’ and ‘Ottaviani’ had smaller fruits than other cultivar/selections, although ‘Hafif Çukurgöbek’ had the highest fruit width and heavier seeds. The fruit length of the ‘Hafif Çukurgöbek’ and ‘Ottaviani’ was shorter than Baduna 5, Güzelyurt-1, and Type 1. The seed numbers were also higher in ‘Hafif Çukurgöbek’ and ‘Ottaviani’ when compared to the others. Seed weight was the lowest in Type 1 resulting in the highest flesh/seed ratio. The ratio in ‘Hafif Çukurgöbek’ was among the lowest. The soluble solids ranged from 10.5 to 12.8%, while the pH ranged from 3.5 to 4.5. ‘Hafif Çukurgöbek’ had the highest pH. There was also a great scale range in acidity, from 0.32 (‘Ottaviani’) to 1.06 (‘Baduna 5’).

**Table 1:** Several pomological characteristics of loquat cultivars grown in Dörtyol, Hatay, Turkey

Cultivar	Fruit weight (g)	Fruit width (mm)	Fruit length (mm)	Seed number/fruit	Seed weight (g/seed)	Flesh/seed ratio	Soluble solids (%)	pH	Acidity (%)
Baduna 5	35.5^a^*	35.4^b^	45.7^a^	2.5^b^	5.7^ab^	5.3^bc^	12.3^ab^	3.5^d^	1.06^a^
Güzelyurt-1	36.1^a^	34.9^b^	44.7^a^	2.3^b^	4.9^bc^	6.4^b^	11.5^ab^	3.6^cd^	0.82^b^
Hafif	30.5^b^	37.2^a^	37.3^b^	3.6^a^	6.7^a^	3.6^c^	12.8^a^	4.5^a^	1.05^a^
Çukurgöbek Ottaviani	27.6^b^	34.5^b^	39.4^b^	3.9^a^	4.4^c^	5.3^bc^	10.5^b^	4.2^b^	0.32^c^
Type1	37.9^a^	34.5^b^	45.7^a^	2.7^b^	3.2^d^	10.9^a^	10.5^b^	3.9^c^	0.65
LSD_0.05_	4.2	1.7	3.4	0.7	1.0	1.9	2.0	0.3	0.22
Mean	33.5	35.3	42.6	3.0	5.0	6.3	11.5	4.0	0.78

* means with different letters are significantly different, at 0.05, by LSD method

**Figure 1:**
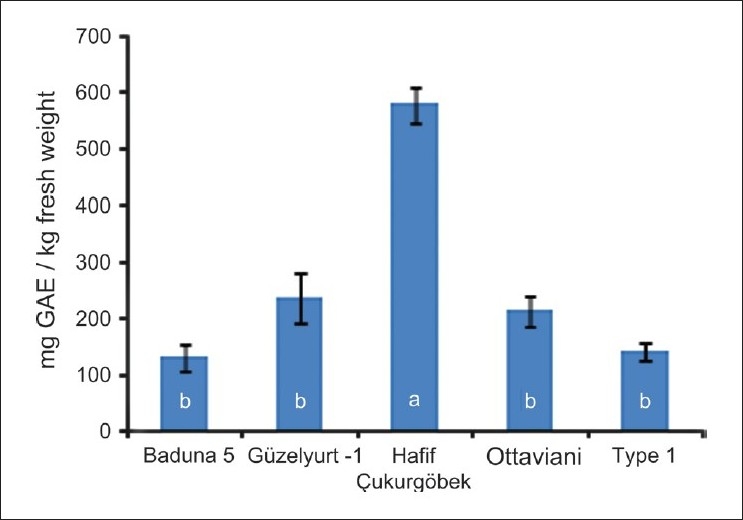
Total phenolic content of several loquat cultivars grown in Dörtyol, Hatay, and Turkey. The bars represent standard deviations and different letters indicate statistically different means determined by the least significance difference at 0.05

**Figure 2:**
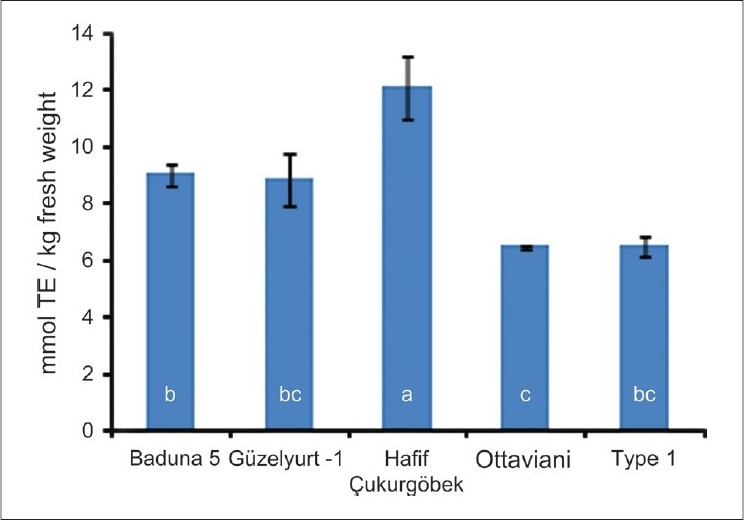
Total antioxidant capacity (Panel B) (determined by FRAP, Ferric Reducing Antioxidant Power) of several loquat cultivars grown in Dörtyol, Hatay, and Turkey. The bars represent standard deviations and different letters indicate statistically different means determined by least significance difference at 0.05

Statistically significant differences were seen for TP [Figure 1]. TP ranged from 129 (‘Baduna 5’) to 578 (‘Hafif Çukurgöbek’) mg Gallic acid equivalent (GAE)/kg fresh fruit (fw). LSD separations indicated that the cultivars/selections were divided into two groups; ‘Hafif Çukurgöbek’ and others. The differences were also significant for TAC, determined by FRAP [Figure 2]. ‘Hafif Çukurgöbek’ also had the highest FRAP mean (12.1 mmol Trolox Equivalent (TE)/kg fw. The others were divided into more complex mean groups as compared to TP. ‘Baduna 5’ had higher FRAP value than ‘Ottaviani’, although Güzelyurt-1 and Type 1 were not different from these cultivars.

The relationships between TP and TAC and among these traits with other pomological characteristics were investigated by correlation analysis [Table 2]. TP and TAC were significantly correlated with the coefficient of 0.73. This is not surprising as these values were found to be correlated with many other fruit species.[17–21] Among the pomological characteristics, fruit width, seed weight, and flesh/seed ratios were significantly correlated with both TP and FRAP. It is also know that within the same fruit species and genotypes, the smaller fruits tend to have higher TP content and TAC, as the compounds resulting in higher activities are usually rich in fruit skin, and smaller fruits have a relatively larger skin area when compared to their large counterparts.[22,23] It is surprising, however, that the flesh/seed ratio was differentially correlated with TP (-0.56) and TAC (0.58). TP was also found to be correlated with fruit length and pH, while FRAP was correlated with soluble solids and acidity.

**Table 2:** Correlation coefficient of several fruit characteristics of loquat cultivars grown in Dörtyol, Hatay, and Turkey

Characteristics	V2	V3	V4	V5	V6	V7	V8	V9	V10	FRAP
Fruit weight (V1)	−0.05	0.76*	−0.71*	−0.20	0.61*	−0.11	0.56*	−0.37	−0.42	0.20
Fruit width (V2)		−0.49	0.23	0.86*	−0.60*	0.38	0.50	0.64*	0.72*	0.61*
Fruit length (V3)			−0.77*	−0.48	0.67*	−0.26	0.79*	−0.10	−0.71*	0.42
Seed number (V4)				0.25	−0.40	0.22	−0.74*	0.32	0.49	0.07
Seed weight (V5)					−0.84*	0.52*	0.30	0.71*	0.66*	0.69*
Flesh/seed ratio (V6)						−0.47	−0.33	−0.33	−0.56*	0.58*
Soluble solids (V7)							0.03	0.62*	0.46	0.59*
pH (V8)								0.19	0.71*	0.31
Acidity (V9)									0.39	0.61*
Phenolic content (V10)										0.73*

* Significant coefficients, at 0.05, are indicated by *, FRAP - Ferric reducing antioxidant power

## CONCLUSION

In this study, we did not determine the carotenoid content and its effect on TAC. More detailed studies are needed to assess the contribution of loquat pigments to TAC. However, our results suggest that loquat cultivars have a variable range of TP content and relatively high total antioxidant capacity, which is crucial for human health.
